# The twin epidemics: Prevalence of TB/HIV co-infection and its associated factors in Ethiopia; A systematic review and meta-analysis

**DOI:** 10.1371/journal.pone.0203986

**Published:** 2018-10-03

**Authors:** Bekele Tesfaye, Animut Alebel, Alemu Gebrie, Abriham Zegeye, Cheru Tesema, Bekalu Kassie

**Affiliations:** 1 Department of Nursing, College of Health Sciences, Debre Markos University, Debre Markos, Ethiopia; 2 Department of Biomedical Science, College of medicine, Debre Markos University, Debre Markos, Ethiopia; 3 Department of Public Health, College of Health Sciences, Debre Markos University, Debre Markos, Ethiopia; 4 Department of midwifery, College of Health Sciences, Debre Markos University, Debre Markos, Ethiopia; University of Cape Town, SOUTH AFRICA

## Abstract

**Background:**

Tuberculosis and HIV/AIDS are the major public health problems in many parts of the world particularly in resource limited countries like Ethiopia. Although studies have been conducted on the prevalence and associated factors of TB / HIV co-infection in Ethiopia, there is no comprehensive data on the magnitude and risk factors at a national and regional levels. Therefore, this review is aimed to summarize the prevalence of TB /HIV co-infection in Ethiopia using meta-analysis based on a systematic review of published articles & grey literatures.

**Methods:**

To conduct this systematic review and meta-analysis, major databases such as Pub Med, Google scholar, CINAHL, Africa Journals Online and Google were systematically searched using search terms. PRISMA guideline was followed in the study. Two authors extracted all necessary data using a standardized data extraction format, and analysis was done using STATA version 11. A Statistical heterogeneity across the studies was evaluated by using Cochran’s Q test and I^2^ statistic. The pooled effect size was conducted in the form of prevalence and associations were measured using odds ratio. Moreover, the univariate meta regression was performed by considering the sample size to determine potential sources of heterogeneity. The Egger’s weighted regression and Begg’s rank correlation tests were used to assess potential publication biases.

**Results:**

This meta-analysis included 21 studies with a total of 12,980 participants. The pooled prevalence of TB / HIV Co-infection was 25.59% (95% CI (20.89%–30.29%). A significant association was found between low CD4 counts (OR: 3.53; 95% CI: 1.55, 8.06), advanced WHO stage (OR: 6.81; 95% CI: 3.91, 11.88) and TB/ HIV/AIDS Co-infection.

**Conclusion:**

This finding revealed that the magnitude of TB /HIV co-infection in Ethiopia is increasing and deserves special attention. Low CD4 count and advanced WHO stage are contributing factors for dual infection. Establishing mechanisms such as Conducting surveillance to determine HIV burden among TB patients and TB burden among HIV patients, and intensifying the three I’s (Intensive case finding, INH Preventive Therapy and Infection control) should be routine work of clinicians. Moreover, early screening & treatment should be provided to those patients with low CD4 count and advanced WHO stage.

## Introduction

The double burden of tuberculosis (TB) and human immunodeficiency virus (HIV) is one of the major global health challenges of the 21^st^ century. TB is the leading immune suppressing infection and the commonest cause of death among HIV-infected patients[1]. According to World Health Organization (WHO), the annual mortality rate due to TB and HIV Co-infection is about 4 million [2]. In 2016, global TB/HIV Co-infection statistics revealed that deaths from HIV and TB co-infection, TB alone, and HIV alone is 374,000, 1,300,000 and 826,000, respectively. Generally, mortality related to TB is greater than HIV infections [3]. Moreover, HIV and TB work together to suppress the immunity of the patients and, thereby, shorten the lifespan if there is no early treatment [4]. WHO estimated that patients living with HIV are 20 times at higher risk of acquiring TB than their counterparts [5]. The incidence of TB is increasing in Africa since HIV is the most contributing factor of people living with HIV/AIDS over the last ten years [6]. In developing countries, the complex factors revolving around TB/HIV infection include stigma, culture, social barriers to testing and treatment, poor health care settings, health-related illiteracy, lack of training, a shortage of medical equipment, lack of manpower, and unqualified laboratory facilities [7].

It is estimated that 1.1 million peoples are living with TB and HIV/AIDS globally and among them 80% are living in Sub-Saharan countries. Similarly, 380,000 people died of both infections. It is the most common clinical feature that even patients that are undertaking antiretroviral therapy (ART) and at least one-third of HIV infected patients had tuberculosis worldwide [8]. The current increase in the global prevalence of HIV infection has an impact on growing global incidence of TB [9]. More than 60% of 15 million cases of dual HIV/TB infection has been reported to Sub-Saharan Africa and the two diseases have been continuing their impact on public health including in South-East Asia [10].

The World Health Organization’s regional director for Europe, Jakab, cautioned that the connection between both TB & HIV/AIDS is inducing high magnitude of drug-resistant TB, which in turn has a potential negative impact on efforts to end TB. She described the complexity of the problem as one-third of people co-infected with TB and HIV often do not aware of their HIV status, and, by extension, are less likely to be cured by either. Moreover, she outlined the consequence as the high rate of disease distribution, weakens the health system and putting governments under stress [5].

Among AIDS related deaths, tuberculosis has the lion’s share of one out of four per year [11]. This linkage of TB/HIV suggests that appropriate prevention strategies have to be established for TB among people living with HIV [11]. Similarly, evidence showed that the risk of acquiring TB increases from the first year of HIV infection [12] and it is associated with low CD4 counts, without timely interventions [12].

Although, the “3 I’s”: intensified TB case finding, isoniazid preventive therapy (IPT), and TB infection control were implemented to decrease the impact of TB among people living with HIV [13] but there is no remarkable achievement especially in Africa.

Sub Saharan Africa region, is an area of high TB/HIV related morbidity and mortality in the world. Therefore, it needs multi-sectoral collaboration to control TB / HIV co-infection [13]. Ethiopia is one of Sub-Saharan countries in which the dual burden of TB/HIV co-infection has severely hit. The Ethiopian Federal HIV and AIDS Prevention and Control Office estimated that the single national HIV/AIDS prevalence was 2.4% in 2010, and the country is among top ten high burden counties with an incidence rate of 341/100,000 of which 31% of TB patients are living with HIV [14]. Although studies have been conducted on the prevalence and associated factors of TB / HIV co-infection in Ethiopia, there is no comprehensive data on the magnitude and risk factors at national and regional levels. Moreover, in Ethiopia there is a low TB case detection and HIV-testing rates that make diagnosis and treatment of TB and HIV more difficult that needs further investigation. Furthermore, it is very critical to the Federal Ministry of Health to advance TB/HIV control program.

## Materials and methods

Both published and unpublished articles done on prevalence of TB/HIV co-infection and associated factors in Ethiopia were retrieved using Preferred Reporting Items for Systematic Reviews and Meta-Analyses (PRISMA) flow diagram. It consists of 27-items intended to facilitate the preparation and reporting of a robust protocol for the systematic review and meta-analysis [S1 File]

### Eligibility criteria and review process

A criterion was established for eligibility before beginning the review of search results. The inclusion criteria were publications in English language and studies conducted in Ethiopia on prevalence and factors of TB / HIV Co-infection from 2007 to 2016. Cross-sectional studies on the prevalence of HIV infection among TB patients and the prevalence of TB among HIV/AIDS patients were used to estimate the pooled prevalence. On the other hand, both case control studies and cross-sectional studies on TB/HIV co- infection were included in this study to identify the associated factors.

### Study selection

The excluded studies were those which did not contain the necessary primary data. Also, those studies which were not published in English language or conducted outside of Ethiopia were excluded. We also removed exact duplicates automatically by using Endnote X7 software after manual review. The primary reviewer (B.T), then, performed a preliminary review by title and abstract to remove articles that were clearly not relevant to the study question or did not meet eligibility criteria. Two reviewers (A.G. and A.Z) independently reviewed the remaining articles in full text, and they each noted whether the article should be included or excluded. If an article had multiple reasons for exclusion, they chose the primary reason for exclusion in the order in which they were listed on the eligibility form. The third reviewer (C.T) was resolved any discrepancy arise during selection process.

#### Information source and search strategy

Major databases such as; Google Scholar, Medline, Psych INFO, Cochrane, Science Citation Index and google was used to find articles. Search terms varied slightly from the controlled vocabulary of the databases except Science Citation Index (which lacks a controlled vocabulary). We chose the terms following a review official medical Heading term in a variety of articles known meeting inclusion criteria. The final search date was December 30, 2017. We included published articles from different databases and unpublished research thesis from Addis Ababa university website. We searched articles by using the above major databases and Addis Ababa official website electronic databases [15]. Articles were searched by using the following key words separately or in a combination of Boolean searches(AND/OR): ("epidemiology"[Subheading] OR "epidemiology"[All Fields] OR "prevalence"[All Fields] OR "prevalence"[MeSH Terms]) AND TB/HIV[All Fields] AND ("co infection"[MeSH Terms] OR "Co-infection"[All Fields] OR ("co"[All Fields] AND "infection"[All Fields]) OR "co infection"[All Fields]) AND determinant[All Fields] AND factors[All Fields] AND ("Ethiopia"[MeSH Terms] OR "Ethiopia"[All Fields]). We also searched the reference lists of the studies that met eligibility criteria, as well as those of review articles about TB/HIV Co-infection.

#### Data collection

The preliminary data abstraction format was prepared by the principal investigator based on outcome of interest and objective of the study. Two authors (B.T and A.G.) independently conducted abstraction of data on the elements such as; authors, publication year, study design, study area, region, age, sample size, response rate, prevalence and factors with respective cross tabulations. Finally, discrepancies were solved through discussion and by inviting the third reviewer.

### Quality assessment

The quality of each study prevalence estimate was assessed by two independent investigators (B.T and A.G.) through disease prevalence quality tool created by Loney et al. [16]. In addition, the quality assessment tool for cross-sectional studies known as Newcastle-Ottawa Scale [17] was used and high quality articles were determined if scale scored is 6 and above out of 10 scales.

### Types of outcome

This study has mainly two outcomes. The primary outcome was the pooled prevalence of TB/HIV co-infection among HIV patients or TB patients in Ethiopia. The second outcome was the associated factors of TB-HIV co-infection. In the included studies, screening of HIV infection was performed by rapid lateral flow immuno-chromatographic test since, assays such as ELISA and Western blot [18] are no longer used in Ethiopia currently. A diagnosis of TB was made based on the combined evaluation of clinical, radiological, histopathological and bacteriological features in accordance with the protocol established by the National Tuberculosis Prevention and Control Program: sample smears/cultures positive, or sample smears/cultures negative but meet all three of the following clinical criteria (1. Symptoms consistent with TB; 2. Chest X-ray suggestive of TB; 3. A positive anti-TB Rx response) [19]. The outcome was measured using the percentage of TB (pulmonary or extra pulmonary) among HIV positive patients and the percentage of HIV/AIDS among TB (pulmonary or extra pulmonary) patients. The association of TB/HIV co-infection and the factors were measured in terms of odds ratio.

### Data analysis

A Microsoft Excel spreadsheet was used to extract the necessary data of each original study using a format prepared by the primary investigator, and then it was imported into STATA 11 statistical software for further analysis. The commands used were metan (for random effects meta-analysis of two variables: double-arcsine-transformed prevalence, Wilson CIs, and prevalence ratios) and metareg (for meta regression). Statistical heterogeneity was evaluated by using Cochran’s Q statistics and I^2^ test, which shows the level of heterogeneity between studies. The percentage of variability due to heterogeneity rather than sampling error or chance differences in effect estimate was determined through I^2^ test. Intrinsically, I^2^ test doesn’t depend on a number of studies incorporated into the study unlike that of Q. Cochran’s Q test verified the presence of heterogeneity (P < 0.10 indicates statistically significant heterogeneity) and I^2^ testmeasured its statistical levels between studies (values of 25%, 50% and75% are to mean low, medium and high heterogeneity respectively) [20]. As the test statistics showed that there was a significant heterogeneity among studies (I^2^ = 97.8%, p = 0.00) i.e. >75%, a random Effects model was used to estimate the summary statistics [17]. The pooled effect size was conducted in the form of prevalence and odds ratio. To minimize the random variations in the point estimates of the primary study, subgroup analysis was done based on the geographical settings. In addition, to identify possible source of heterogeneity, univariate meta regression was performed by considering the sample size of each study, but the finding was not statistically significant. Point prevalence with their 95% confidence interval was presented in forest plot. In this plot, the size of each box indicated the weight of the study, while each crossed the line refers to 95% confidence interval.

The Egger’s weighted regression and Begg’s rank correlation test methods were also used toassess publication bias (P < 0.05 was considered as suggestive of statisticallysignificant publication bias).

## Results

**Fig 1:**
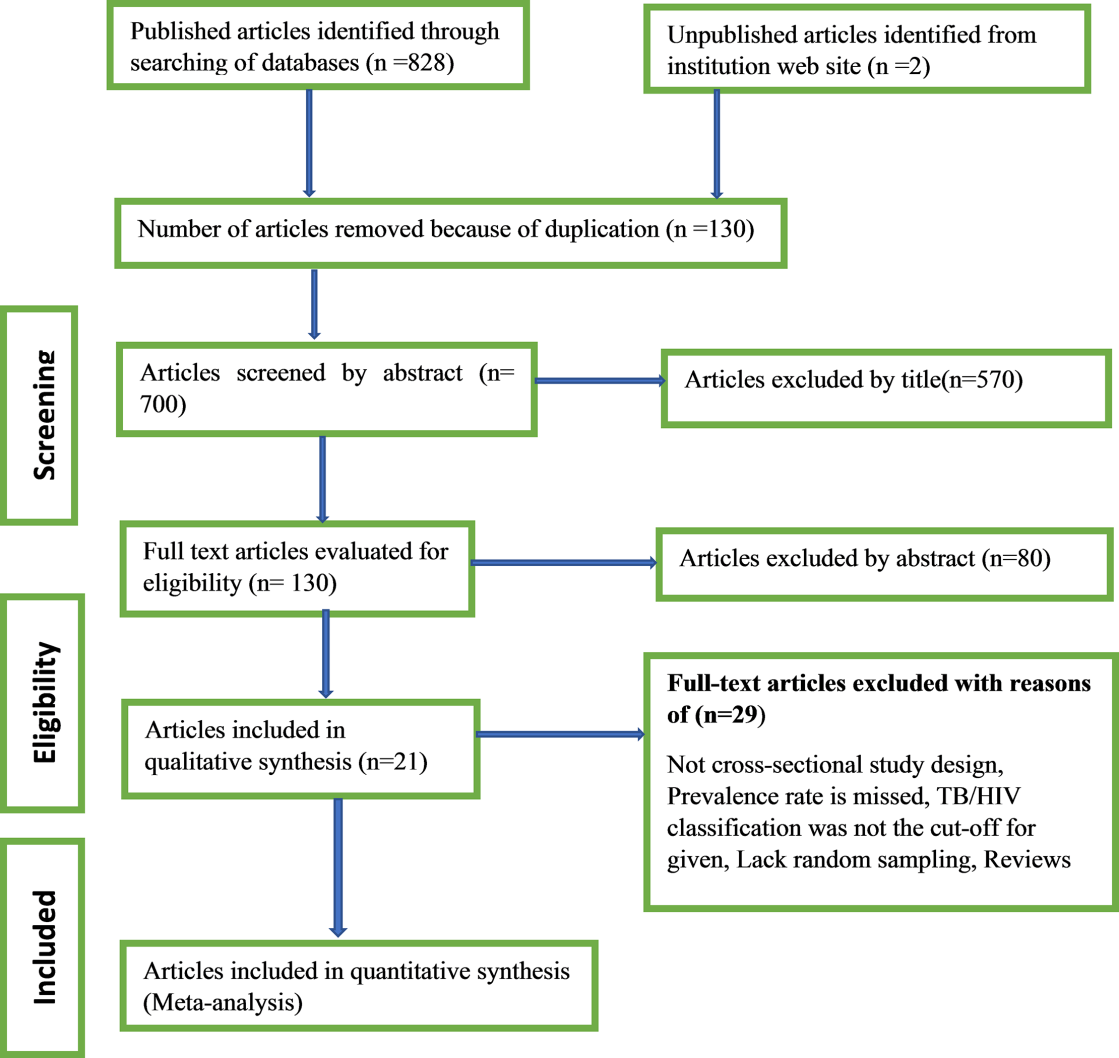
Flow diagram of the studies included in the meta-analysis on TB/HIV co-infection.

We identified 830 articles in the electronic search of the different databases as mentioned previously, google for unpublished literatures/ one study from Addis Ababa university online digital library/, and reference lists of previous studies. From 830 retrievals, 570 of them were excluded after reviewing their titles, 130 by reviewing of abstracts, and the remaining were excluded due to different reasons as shown below. Finally, 21 studies were included in the meta–analysis [Fig 1].

### Characteristics of included studies

All 21 included articles are observational studies with cross-sectional and case control study design. Fourteen of them were institution/hospital based studies [21–36] whereas five of them were community (population) based studies [37–41]. Two of them were unpublished articles [22, 32]. The sample sizes of the studies range from a minimum of 275, a study conducted at Gondar University Hospital, Northwest Ethiopia to a maximum of 2886, a study conducted from January 2011- December 2015 in the Directly Observed Treatment, Short Course (DOTS) clinic of Debre Markos Referral Hospital, North west Ethiopia. A total of 12,980 participants were included in the review. The studies were conducted from 2007 to 2017 in different five regions of the country such as; Amhara, Afar, Oromia, Southern nation and nationality, and Addis Ababa city administration. Fifteen articles/cross sectional studies/ were analyzed for prevalence (Table 1) and the remaining six/case control studies/ were used for analyzing associated factors such as; low CD4 count and advanced clinical stages in addition to five studies from cross sectional studies.

**Table 1:** Summary characteristics of studies in the meta-analysis to show the prevalence TB/HIV co-infection in Ethiopia.

S.No	Authors	Publication Years	Study setting	Study Area	Region	Age	Total Sample	Included Sample	Outcome (Frequency)	Response Rate	Prevalence
1	Atnafu Temesgen	2016	Hospital Based	Debre Markos Referral Hospital	Amhara	< = 15 & > = 60	2886	2886	543	100.0	18.8
2	Tarekegne et al	2017	Hospital Based	Metema Hospital	Amhara	< = 14 & > = 65	2096	2005	404	95.7	20.1
3	Asnake Simieneh et al	2017	Hospital Based	Hawassa University Referral Hospital	SNNP	Non-specific	1961	1858	246	94.7	13.9
4	Tadesse Andtadess et al	2013	Facility Based	Dabat 2 Health Centers	Amhara	0–14& > = 40	1081	849	97	78.2	11.4
5	Mekonnen et al	2015	Facility Based	Kobo, Robit And Gobiye	Amhara	< = 24& > = 46	991	972	236	98.2	24.3
6	Ahmed Esmael et al	2013	Hospital Based	Debre Markos Referral Hospital	Amhara	< = 15 & > = 31	717	717	321	100	44.8
7	Deribew et al	2014	Facility Based	Adama, Nekemet And Jimma Specialized Hospitals	Others	> = 15	620	591	124	95	20.9
8	Aweke et al	2016	Facility Based	5 Hospitals in Amhara Region	Amhara	>15	571	571	158	100	27.7
9	Yasin et al	2015	Hospital Based	5 Rural Hospitals in South	SNNP	Any Age	500	500	97	100	19.4
10	Fekadu et al	2015	Hospital Based	Hawassa University Referral Hospital	SNNP	> = 18 Month	499	499	91	100	18.2
11	Wondimeneh et al	2012	Hospital Based	Gonder Hospital	Amhara	> = 18	422	400	30	94.8	7.5
12	Animut Ayenew et al	2010	Hospital Based	East Gojjam Hospital	Amhara	> = 15	355	283	128	79.7	36.2
13	Mulugeta Belay et al	2015	Facility Based	Awash Health Center and Dubti Hospita)	Others	> = 18	325	287	82	88.3	28.6
14	Esayas Kebede Gudina et al	2013	Hospital Based	Jimma University	Others	Ns	287	287	129	100	45
15	Afework Kassu et al	2007	Hospital Based	Gonder Hospital	Amhara	> = 18	275	275	134	100	52.1

NB

• Others (Oromia, Addis Ababa & Afar regions)

• SNNP = Southern Nation and Nationalities Peoples Region

### Quality of studies included in the systematic review

The quality score of studies ranged from three to seven out of ten; the majority of studies, their statistical quality and data presentation methods were poor to medium level. In addition, all of the studies used a binary logistic regression model on analysis to identify the association of different risk factors with TB/HIV co-infection. Furthermore, chi-squared test was used when comparing groups. Only four studies showed the association for low CD4 count [21, 26, 33, 40] and five studies showed the association of Advanced WHO clinical stage [26, 33, 34, 36, 42] with the outcome variable (TB/HIV co-infection). Three studies used Student’s t test to compare means to normally distributed variables [21, 31, 40]. Two studies evaluated goodness of fit to normal distribution using the Hosmer and Lemeshow goodness of fit test [21, 31]. But, none of the studies checked for multi-collinearity through VIF/tolerance, and the standard errors of regression coefficients.

### Heterogeneity and publication bias

**Fig 2:**
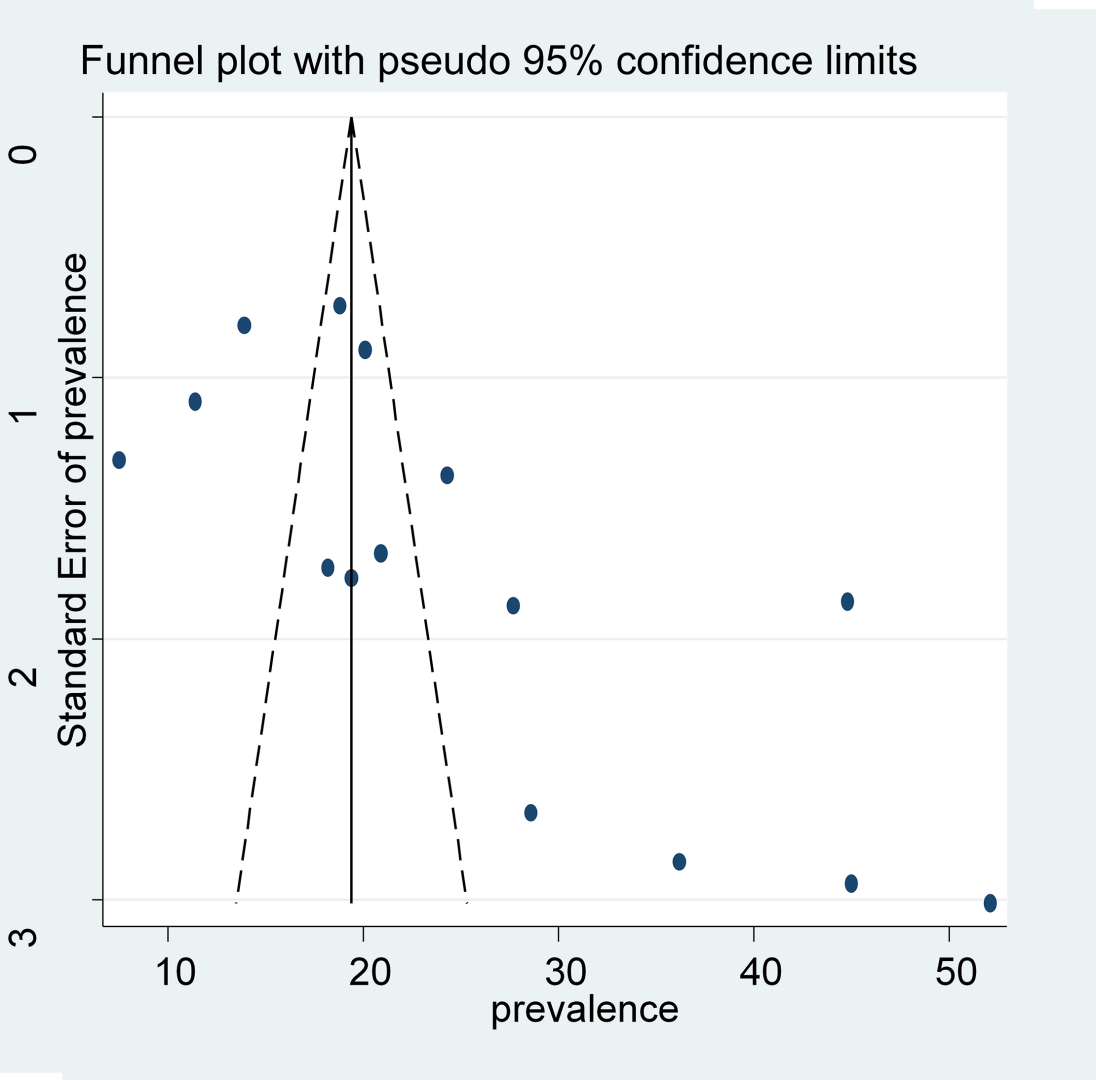
The funnel plots are asymmetric and indicates possible publication bias.

The level of statistical heterogeneity across studies was measured by using I^2^ test and the presence of heterogeneity was determined through Cochran’s Q test (P < 0.10 indicates statistically significant heterogeneity). As the test statistic showed there was a significant heterogeneity among studies (I^2^ = 97.8%, p = 0.00) as a result random Effects model was used to estimate the Der Simonian and Laird's pooled effect. The Egger’s weighted (regression, p = 0.02) and Begg’s rank correlation test (p = 0.03) methods were used to assess publication bias, and since p< 0.05 suggests statistically significant publication bias. Duval and Tweedie’s trim and fill methods were used to estimate the number of studies missed from a meta-analysis as a source of publication bias but the finding is not significant [43]. The funnel plots of TB/HIV co-infection were asymmetric, indicating a possible publication bias [Fig 2].

### Meta-analysis

#### Prevalence of TB/HIV co-infection in Ethiopia

**Fig 3:**
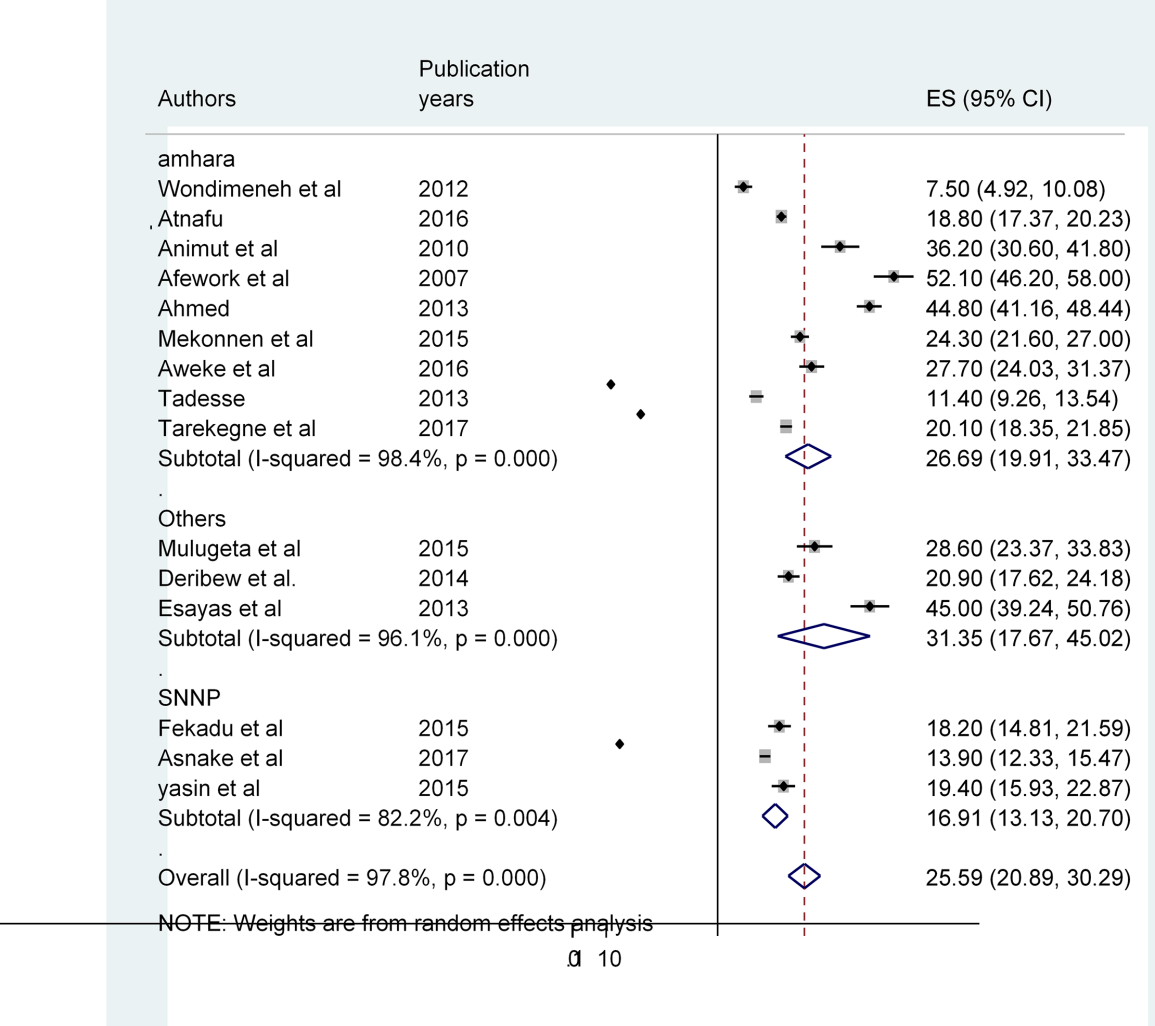
The Pooled prevalence of TB/HIV co-infection among different regions in Ethiopia/sub group Analysis.

Among the 21 articles included in this study, the lowest magnitude (7.5%) was recorded among adult HIV-positive patients attending HIV/AIDS clinic of Gondar University Hospital [44] and similarly, the highest (52.1%) was at Gondar town health facilities, Northwest Ethiopia [45]. The random effect model was used since there is high heterogeneity (I^2^ = 97.8%) across studies (Fig 2). The comprehensive (pooled) prevalence of TB/HIV co-infection in Ethiopia was 25.59% (95% CI (20.89%–30.29%). We conducted further analysis across regions of the country (sub-group analysis) separately, and it revealed that the magnitude of dual infection is different between regions [Fig 3]. The prevalence in Southern Nation and Nationalities region is 16.1% (95%CI (13.13, 20.70), in other regions such as; Addis Ababa and Oromia is 31.35, 95% CI (17.67%,45.02%) and in Amhara region it is 26.69%, 95%CI (19.91%, 31.47%).

#### Associated factors of TB/HIV co-infection in Ethiopia

**Fig 4:**
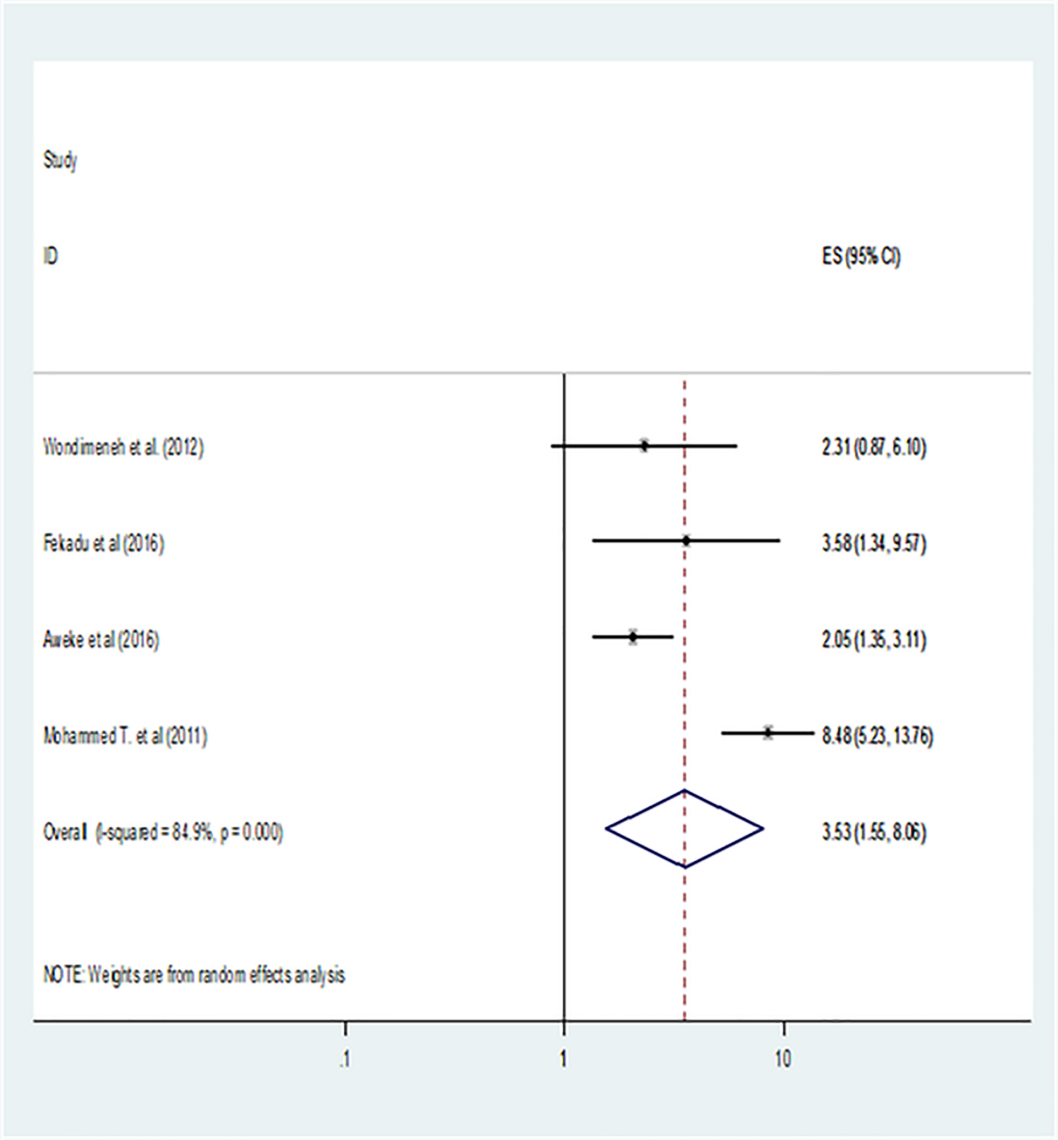
The association between low CD4 count and TB/HIV co-infection in Ethiopia.

**Fig 5:**
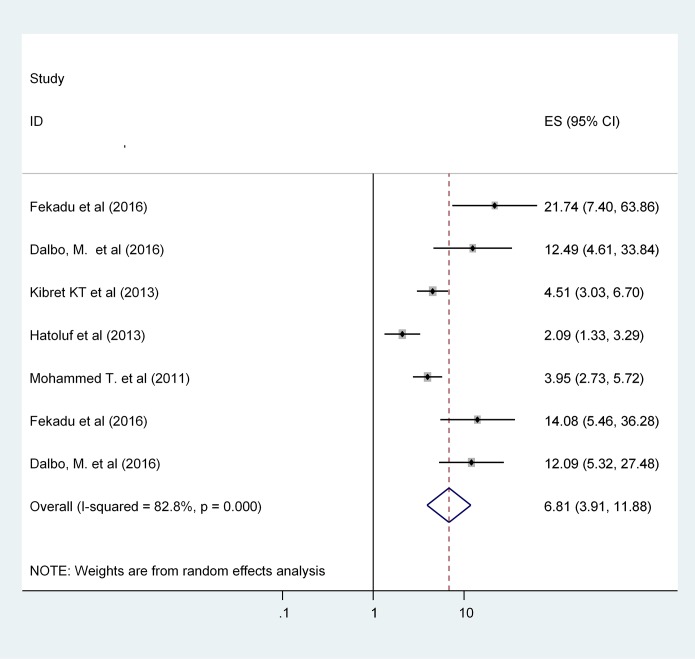
The association between Advanced WHO clinical stage and TB/HIV co-infection in Ethiopia.

A total of 11 (five from cross sectional and 6 from case control studies) articles has been included to identify factors that were associated with the TB/HIV Co infection. This study identified two main factors namely: low CD4 count (less than 200 cells/mm^3^) and advanced WHO clinical stage (WHO stage 3 & 4). Four and seven articles were separately analyzed for low CD4 count and advanced WHO clinical stage, respectively. Patients with low CD4 count are 3.5 times more likely to have TB/HIV co-infection as compared to high CD4 count (OR: 3.53, 95% CI: 1.55, 8.06) [Fig 4]. Patients with advanced WHO clinical stage were 6.81 times more likely to have TB/HIV co-infection as compared to WHO stage one (OR: 6.81, 95% CI: 3.91, 11.88) [Fig 5].

## Discussion

The dual TB/HIV infection in different part of Ethiopia is addressed in this review. The interaction between TB and HIV is bidirectional and is a dual public health burden. In this review the magnitude of TB/HIV co-infection was higher among HIV/AIDS patients than TB patients. People with HIV are more likely to have TB compared to people without HIV. TB in turn increases the HIV/AIDS disease progress through chronic stimulation of the immune [36, 46].

This study revealed that TB / HIV co-infection is well recognized as a major public health problem in Ethiopia and it is in line with the findings of western countries [47–49]. The result also supports the report on the United Nations Joint Program on HIV/AIDS (UNAIDS) in which one-third of them are co- infected with TB [50]. This is because of the fact that TB is one of opportunistic infections which commonly take advantage of weakened immunity due to HIV.

Moreover, this finding is also congruent with studies conducted in China, India and Brazil in which the pooled prevalence is 23.5%, 18.9% and 19%, respectively [51–53]. Furthermore, the prevalence of this study is similar to the study findings in African countries, 31.25%, in European countries, 20.11%, in Latin America, 25.06% [51]. This indicates that the dual infection is still public health issues, even in the industrialized countries. But the finding of this study is higher than studies conducted in five districts of the Volta Region of Ghana (18.1%) [54], Pacific Asian countries (17.2%) [55], global estimate (15%). This discrepancy, in Pacific Asian countries, might be due to the limitation of data on the rate of HIV infection among TB patients and only small countries test TB patients for HIV [28]. On the other hand, the variation on monitoring systems and collaboration on TB/HIV control programs across countries may have contributed its fair share.

This finding is lower than the WHO estimate for African Region (36%) [56]. This might be due to a limitation of current TB tests to diagnose TB in PLWHA compared to people without HIV infection and results in many undiagnosed TB cases. On the contrary diagnosing HIV in TB patients is easy because of inexpensive and quick HIV tests which results in the better estimation of HIV among TB patients [57].

The current finding is also higher than the prevalence reported for Asian countries (17.21%), USA (14.84%), a rate (13%) reported by WHO in 2011 [51, 58]. This might be due to the fact that Asian countries have better awareness about TB/HIV co-infection and its treatment than African countries including Ethiopia. On the other hand, in the USA especially, in California the dramatic decline is due to the effectiveness of HAART and TB control [59]. Even though the TB/HIV co-infection in industrialized nations [60] is lower than Sub-Saharan Africa [61]there is increment in some part of their regions. For instance, in the united states [62] it is increasing; as a result of the demographic transformation of TB/HIV by immigrants of countries such as; Mexico/ Central America and Southeast Asia citizens.

The pooled prevalence in this study is consistent with the report on Canada [63] and sub- Saharan Africa [64] in which 33% and 70% of all people living with HIV are co-infected with both TB and HIV respectively. This increment in magnitude, especially in developing countries, is due to the reason that there is inconsistent action to tackle the problem of resource-limited areas like Sub-Saharan countries. This needs the attention of international donor agencies, such as WHO and USAIDS joint approach including testing and treatment for dual infection. Moreover, there is a variation across the countries to address TB-HIV co-infection because of poorly tracked and incomprehensible strategy, poor collaboration between donor countries and multilateral institutions to address the TB/HIV infection [65]. On the other hand, in Ethiopia, there is a slight decrement of TB/HIV dual infection as compared to the above countries due to the fact that the Ethiopian Ministry of Health endorsed health extension program in which two community health trained practitioners work at health posts in every rural community between 500 and 1,500 households with more than 30,000 health extension workers [66].

The program is supported by government, global fund grants and donor funds for TB/HIV collaborative service such as; HIV testing, counseling, Antiretroviral therapy, and anti-TB therapy for TB/HIV patients and thereby reduces the prevalence of dual infection.

In this study there are regional disparities in the country in sub-regional analysis. This might be due to the fact that urban versus rural residence vary from regions since urban population are more vulnerable than rural populations to dual infection. Furthermore, high risk populations such as; commercial sex workers, militaries, long track drivers, police officers, unfair distribution of healthcare professionals across regions due to unsuitable topography of the country, poor access to sparse health services, inefficient procurement systems, and weak monitoring and evaluation systems [67] results in regional variation. On the other hand, famine, conflict and drought leads to cross border tensions in refugee camps which results in an increment of the infection around these areas [48]. Furthermore, the TB/HIV co-infection situation in Ethiopia continues to be characterized by a low-intensity, mixed epidemic with significant heterogeneity across geographic areas and defined by independent, self-sustaining HIV transmission streams within key Population, priority population, and general populations [68]. Per spectrum preliminary estimate by the U.S. President's Emergency Plan for AIDS Relief (PEPFAR) [69], January 2017, adult TB/HIV prevalence in Ethiopia in 2016 was estimated to be 1.1%. There is substantial prevalence variation by region (6.6% in Gambella, 5.0% in Addis Ababa, and 0.7% in Southern Nations, Nationalities and Peoples’ (SNNPR) region). The TB/HIV epidemic in Ethiopia is primarily associated with areas of urban concentration (5.1% in cities above 50 thousand compared to 3.1% in smaller cities and 0.6% in rural areas) and proximity to major transport corridors [69]. Those living within five kilometers of a major road have TB/HIV prevalence rates that are four-times higher than those who live further away. The two exceptions to this general pattern include Gambella region, a small and sparsely populated region that has the highest regional prevalence in Ethiopia (6.4%) and little distinction between urban and rural prevalence, and development schemes and seasonal migrant destinations that show elevated HIV-related risk behaviors despite not being close to urban areas or major roads. Another defining feature of the Ethiopian TB/HIV epidemic is the pattern of steep and steady declines in antenatal clinic (ANC) prevalence by as much as 60% since 2005 when PEPFAR and the Millennium AIDS Campaign signaled the start of a robust and successful national response [68, 69].

The other key areas of regional variation across the country relate to the ministry of health AIDS surveillance system. The nationwide data are mainly based on monthly reports on hospitals, ANC sentimental surveillance, blood donors and some special surveys. But, both hospitals and ANC services are not accessible for rural populations living with HIV & mainly restricted in Addis Ababa and regional towns. For instance, the nationwide instruments of the national rates, ANC attendees at 13 hospital and clinic sentinel sites were significantly variable [70] and results in discrepancies across regions.

In the current study, the important factors associated with TB/HIV co-infection were low CD4 count (<200cells/mm^3^) and advanced WHO stage (3 & 4). On the contrary, studies in Sub Saharan Africa countries [10, 54, 71, 72] and a single study in Northwest Ethiopia [73] revealed sex as a significant factor. The reasons reported to female patients were; lower socioeconomic status of women, biology, sexual behavior, the gender difference in access to resources and decision-making power variation [9, 73, 74]. In this study one of the common factors is CD4 lymphocyte, which is a type of white blood cell that is important to immune function. The lower the number of CD4 cells, the more impaired the immune system will be and it is a good indicator of the health of the immune system in PLHIV [75]. Similarly, the atypical radiographic appearance of pulmonary TB is common in AIDS patients with CD4 T-lymphocytes count less than 200 cells/ μL [76].

According to WHO, People Living With HIV (PLWHIV) who had low CD4 counts are at greater risk of developing TB infection [77]. On the other hand, the second common factor is an advanced clinical stage, which is developed for health care professionals to estimate the severity of immune deficiency in HIV patients. Staging simply categorizes PLWHIV to one of four categories of WHO stages. Stage one has no serious immune deficiency and signs of opportunistic infections. Stage 3 or 4 has a severe immune deficiency and signs of moderate and severe opportunistic infections. Among this, TB is the single most important opportunistic infections that show immune suppression [75]. Generally, this study showed the concept that AIDS is the sum total of laboratory diagnosis plus an opportunistic infection or a CD4 count of below 200/μL.

### Limitations of the review

This study analyzed the prevalence and associated factors of both tuberculosis and HIV/AIDS patients by using multiple databases to search articles (both manual and electronic searches) for meta-analysis.

The main limitations of this study include the low number of studies from rural areas on prevalence and associated factors in Ethiopia available for analysis. Even though we included articles in different parts of the country, still the representativeness of the population is not as such strong. Moreover, this study analyzed population-based prevalence of TB/HIV co-infection but not specific prevalence among TB patients at TB clinics and among people at Antiretroviral clinic.

## Conclusions

This finding revealed that the magnitude of TB /HIV co-infection in Ethiopia is increasing and deserves special attention. Low CD4 count and advanced WHO stage are contributing factors for the dual infection. Establishing mechanisms such as Conducting surveillance to determine HIV burden among TB patients and TB burden among HIV patients, and intensifying the three I’s (Intensive case finding, INH Preventive Therapy and Infection control) should be routine work of clinicians and other concerned bodies. Furthermore, early screening & treatment should be provided to those patients with low CD4 count and advanced WHO stage.

## Supporting information

S1 File PRISMA 2009 checklist. (DOC)
